# Outcomes following transcatheter transseptal versus transapical mitral valve-in-valve and valve-in-ring procedures

**DOI:** 10.15171/jcvtr.2018.31

**Published:** 2018-12-09

**Authors:** Salik Nazir, Saroj Lohani, Niranjan Tachamo, Muhammad Sohail Khan, Bidhya Timilsina, Faraz Khan Luni, Anthony Donato

**Affiliations:** ^1^Department of Medicine, Reading Hospital-Tower Health System, West Reading, Pennsylvania, USA; ^2^Division of Cardiology, Mercy Saint Vincent Medical Center, Toledo, Ohio, USA; ^3^Sidney Kimmel Medical College at Thomas Jefferson University, Philadelphia, Pennsylvania, USA

**Keywords:** Transcatheter, Transapical, Transseptal, Mitral Valve

## Abstract

***Introduction:*** Transcatheter mitral valve-in-valve (ViV) & valve-in-ring (ViR) are relatively novel therapeutic alternatives for patients with degenerated bioprostheses or failed annuloplasty rings whose reoperative risk is too high. The predominant procedural access for both procedures is transapical or transseptal. However, whether there are differences in outcomes of this procedure using transseptal versus transapical access has not yet been defined.

***Methods:*** We conducted a systematic review of all published articles from MEDLINE and EMBASE to explore the outcomes of these two procedural approaches.

**Results:**total of 55 studies including 183 patients (154 ViV and 29 ViR) were included. Patients that underwent ViV (101 transapical and 53 transseptal) using the transseptal approach required more iatrogenic atrial septal defect (ASD) closure (19% versus 0.0 %; *P* < 0.001) and hence had a lower device success rate (68% versus 89%; *P* = 0.001). However, there was no significant difference between the two groups in procedural success and all-cause mortality at 30 days. Overall severe bleeding complications (major or life threatening) were not different the two groups (3.7% versus 7.9%; *P* = 0.321). In the ViR group (19 transapical and 10 transseptal), no difference in procedural success, device success or 30-day outcomes were identified between transseptal and transapical groups, although sample size was small.

***Conclusion:*** In conclusion, mitral ViV and ViR using the two different procedural approaches appear to confer equal and reasonable 30-day outcomes.

## Introduction

Transcatheter mitral valve-in-valve (ViV) and valve-in-ring (ViR) procedures are relatively novel therapeutic alternatives for patients with degenerated bioprostheses or failed annuloplasty rings whose risk of surgical reoperation is deemed to be too high.^1,2^ While transapical access was the first described approach,^3^ this procedure requires a thoracotomy with its attendant risks. As implantable valve technology has evolved, so has the interest in implantation through venous access, performed by accessing the mitral orifice through a puncture in the atrial septum (transseptal) approach. Data from the United States Society of Thoracic Surgeons/American College of Cardiology Transcatheter Valve Therapy (TVT) registry showed that transseptal placement increased from 14.6 % to 28.2 % in 2015.^1^ This trend bears some resemblance to procedural trends in transcatheter aortic valve replacement, in which initial experience was with transthoracic deployment approaches, however was soon supplanted by transfemoral placement as reduction in sheath size allowed for placement via peripheral arterial access.^1,4^

Benefits of transapical placement include better control over the implant position and possibly less device-related complications, while transseptal placement avoids a thoracotomy but involves puncture of the atrial septum and hence possibility of the need to later repair an iatrogenic atrial septal defect (ASD).^5^ Furthermore, registry data from the VIVID (Valve-in-Valve-International-Data) showed that patients having transseptal procedures had a statistically significant improvement in myocardial contraction in patients with left ventricular dysfunction compared to those treated transapically.^5^ Clearer understanding of the immediate and subacute outcomes are needed for these two procedural approaches to help guide interventional cardiologists about the risks and benefits associated with each approach.

The purpose of this study was to evaluate and synthesize the published outcomes of transseptal versus transapical mitral ViV and ViR procedures to date.

## Materials and Methods

Systematic electronic search of MEDLINE and EMBASE databases were performed for case reports and case series on transcatheter mitral ViV and ViR procedures from inception until June 26, 2017 using a broad series of search terms (Supplementary file 1). We also performed a hand search of references from included articles to identify additional publications. Authors were contacted as well if additional information was required.

### Inclusion criteria

1. Articles in English
2. Minimum of 30 days follow up post-procedure

### Exclusion criteria

1. Combined valvular procedures (such as transcatheter mitral valve implantation and aortic valve implantation)
2. Procedures performed through left atrial approach

Screening of articles for eligibility was performed by two authors (SN, SL). Conflicts during the screening process were resolved by third author (NT). Data extraction was performed by five authors (SN, SL, NT, MSK, BT) and discrepancies were resolved by two authors (SL, NT). Data extracted on the cases included indications for surgery, risk scores, etiology of valve dysfunction, procedural approach, pre-and post- procedure mitral valve gradient measurements, pre- and post-procedure ejection fractions, ICU and total length of hospital stay, procedural success, device success and complications. Risk scores were collected as reported in the individual papers and were not calculated individually. Primary end-point of the present study was all cause mortality at 30 days. Secondary endpoints were device and procedural success and other complications defined according to the Mitral Valve Academic Research Consortium (MVARC) criteria.^6,7^ Device success was assessed at 30 days and at later post -procedural intervals. Device success was defined as all of the follows: absence of procedural mortality or stroke; proper placement and positioning of the device; freedom from any unplanned surgical or interventional procedures related to the device or access procedure; continued intended safety and performance of the device, including: (1) no specific device-related technical failure issues and complications; (2) no evidence of structural or functional failure; and (3) reduction of mitral regurgitation to either optimal or acceptable levels (no more than moderate +2 in severity) and without significant mitral stenosis. Although the MVARC criteria defined significant mitral stenosis as a post procedure effective orifice area (EOA) <1.5 cm^2^ or a transmitral gradient ≥5 mm Hg, a post-procedure transmitral gradient ≥5 mm Hg was commonly reported in studies of mitral ViV and ViR.^2,8^ Therefore, for our study we used American Society of Echocardiography guidelines criteria for significant mitral stenosis, which is defined as EOA ≤1 cm^2^ or a transmitral gradient ≥10 mm Hg.^9^ Procedural success was determined at 30 days and it was defined as a procedure that achieved device success without major clinical complications (stroke, acute kidney injury 2 or 3, new requirement for dialysis, stroke, life-threatening/fatal bleeding, major vascular complications, death, valve-related complications or other complications requiring surgery or repeat interventions. Categorical variables are expressed as percentage and continuous variables as mean ± SD. Student *t* test and chi-square were used for statistical analysis, using Microsoft Excel 2010 (Microsoft Corporation, Redmond, VA) and Stata 13.1 (Stata Corporation, College Station, TX).

## Results

**Figure 1:**
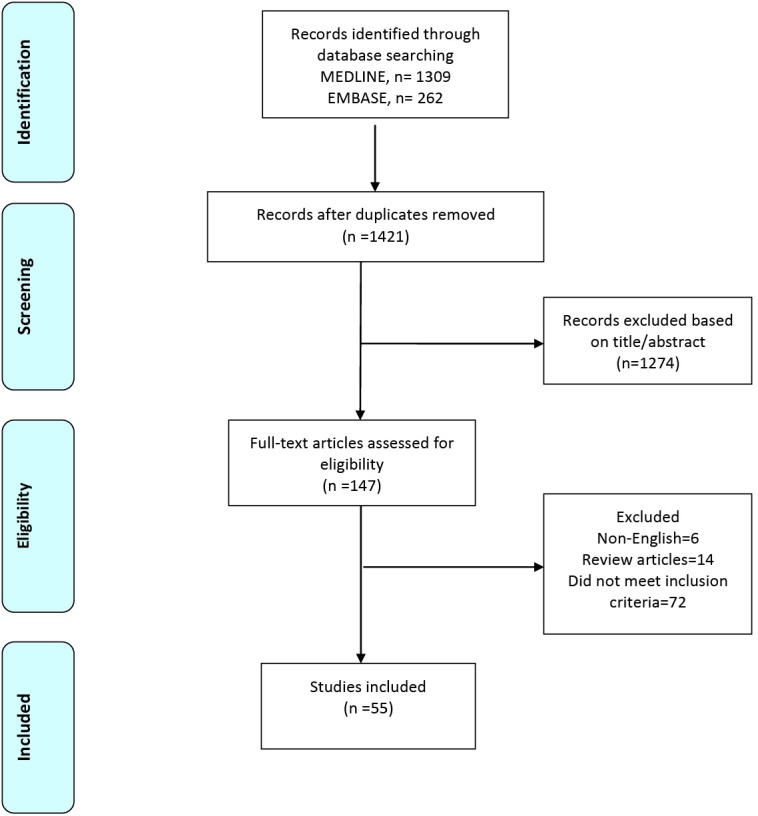
Flow chart describing systematic search and study selection process.

We identified a total of 183 cases (154 ViV and 29 ViR) from 55 published articles (Figure 1). Co-morbidities, risk scores, echocardiographic parameters, type of valves, complications and outcomes were abstracted (Supplementary file 2, Tables 1 and 2). The mean age of patients was (76 ± 8.6 years). Society of Thoracic Surgeons (STS) risk score were reported in 157 cases, which predicted a 30-day mortality of 15 ± 11.5 %, while logistic euroSCORES were reported in 91 cases and predicted an estimated surgical risk of 34.5 ± 16.5 %. In terms of primary prosthesis failure modes, etiology included regurgitation in 75 cases (41% of total), stenosis in 45 cases (25% of total), mixed stenosis and regurgitation in 30 cases (16 % or total) and not reported in 34 cases (18% of total). The mean length of ICU stay was 5.2 ± 10 days (range: 1-47 days) and the mean length of hospital stay was 11 ±12 days (range: 2-70 days). The mean mitral valve gradient was significantly lower post-procedure compared with pre-procedure (5.6 ± 2.39 mm Hg versus 11.5 ± 5.67 mm Hg, *P *< 0.001). The mean ejection fraction pre- and post-procedure was reported in only 7 patients and showed no significant difference (52 ± 11.5 % versus 54.8 ± 9.2 %, *P *< 0.634). A total of 154 patients underwent ViV procedure. Transapical access was used in 101 (65.5%) cases whereas transseptal was utilized in 53 (34.4%) patients. Patients who underwent ViV using the transseptal approach required more iatrogenic ASD closure (19 % versus 0.0 %; *P *< 0.001) and hence had a lower device success rate (68% versus 89%; *P *= 0.001). However, there was no significant difference between the two groups in procedural success and all-cause mortality at 30 days. Overall bleeding complications (major or life threatening) were not different the two groups (3.7% versus 7.9 %; *P *< 0.321). Life- threatening or fatal bleeding was reported in one patient in the transapical group^10^ and two patients in the transseptal group.^11^ However, in both these patients, a transapical rail was also used along with the transseptal approach. Key changes were made in the procedure by the authors, including not utilizing transapical rail, which then led to higher rates of procedural success and lower complications. Left ventricular outflow tract (LVOT) obstruction was observed in one patient^12^ in the transapical group which was managed conservatively. The outcomes of ViV using two different procedural approaches are depicted in Table 1.

**Table 1:** Outcomes & complications of transseptal versus transapical mitral valve in valve procedure

	**Transseptal (n=53)**	**Transapical (n=101)**	***P*** ** value**
ASD closure	10 (19 %)	0	<0.001
LVOT obstruction	0	1 (0.9%)	0.467
AKI (Stage 2 or 3)	0	3 (2.9 %)	0.205
AKI requiring hemodialysis	1 (1.8 %)	7 (6.9 %)	0.180
Overall bleeding complications	2 (3.7 %)	8 (7.9 %)	0.321
Major/extensive	0	7 (6.9 %)	0.050
Life threatening/Fatal	2 (3.7 %)	1 (0.9 %)	0.235
Stroke/TIA	0	1 (0.9 %)	0.467
Need for permanent pacemaker implantation	0	2 (1.9 %)	0.302
Length of hospital stay	13 days (SD 13.6)	11.4 days (SD 12.7)	0.757
Procedural success	36 (68 %)	82 (81%)	0.065
Device success	36 (68 %)	90 (89%)	0.001
All-cause mortality at 30 days	5 (9.4%)	7 (6.9%)	0.582

A total of 29 patients underwent ViR procedure. Transapical access was utilized in 19 (65.5%) cases and transseptal was used in 10 (34.5 %) cases. In the ViR group, no difference in procedural success, device success or 30-day outcomes were identified between transseptal and transapical groups, although sample size was small. LVOT obstruction developed in two patients in the transseptal group^11^ and two patients in the transapical group.^13^ In the transseptal group, the LVOT obstruction was managed by surgical resection of the anterior mitral leaflet in one patient, while the other was managed conservatively. In the transapical group, one patient required recapturing of the device and in the other patient LVOT obstruction was eliminated by repositioning of the valve. The outcomes of ViR using the two different procedural approaches are depicted in Table 2.

**Table 2:** Outcomes of transseptal versus transapical mitral valve in ring procedure

	**Transseptal (n=10)**	**Transapical (n=19)**	***P***
ASD Closure	0	0	-
Life threatening or Fatal Bleeding	0	0	-
Major or Extensive Bleeding	0	0	-
Stroke/TIA	0	2 (10.5%)	0.288
Need for Permanent pacemaker implantation	0	1 (5.2%)	0.460
LVOT Obstruction	2 (20%)	2 (10.5%)	0.482
Procedural success	7 (70%)	14 (73.6%)	0.833
Device Success	7 (70%)	14 (73.6%)	0.833
All-cause mortality at 30 days	0	3 (15.7 %)	0.184

## Discussion

Our review showed patients that underwent ViV and ViR procedures were largely elderly, and carried a high predicted surgical risk of 30-day mortality. This is in line with previously published data that showed the utility of these procedures mainly in patients considered at high risk of open heart surgery.^11,14,15^ In the ViV group we identified that transseptal procedures required an additional procedure to close their iatrogenic ASDs created during valve placement, a factor which lowered its device success rate but did not affect its overall procedural success or 30-day all-cause mortality rates. In a recent analysis from transcatheter mitral valve replacement (TMVR) multicenter registry, Sung-Han et al^2^ also identified the need for increased ASD closure procedures following valve implantation in the transseptal group as compared to transapical group but no difference in procedural outcomes and 30- day mortality in the combined cohort of ViV and ViR.

Our review identified a non-significantly higher rate of bleeding in patients undergoing transapical placement as compared to transseptal (3.7 % versus 7.9 %; *P *= 0.321). Given that two of the transseptal bleeds involved a variant of the procedure (a trans-apical rail), an approach that was later abandoned, the risk of bleeding with the transseptal approach has likely lessened. The TMVR trial noted a 6% rate of major/extensive and a 2.3% rate of life-threatening or fatal bleeding in their ViV patients, but did not break this down by type of procedural access.

In the present study, the outcomes of ViR did not differ between the two procedural approaches likely because of the limited sample size. In the study by Sung-Hen et al^2^ the authors reported lower procedural success, more frequent life- threatening or fatal bleeding and increased all-cause mortality at 1 year in patients that underwent ViR compared to ViV. The individual outcomes of ViR using transseptal versus transapical access were not reported separately in this study.

There are several limitations to this study. The studies collected were case reports or case series with limited numbers and therefore, the results may be subject to publication bias. Cases with unexpected or undesired results are less likely to be published hence the outcomes in this study could differ from those in real-world practice. In comparing the complications of the different procedural approaches, we were not able to control for operator procedural experience or hospital experience that may have affected complication rates. The quality of the synthesis of the data for our study was dependent on each study uniformly reporting study parameters and outcomes, and was therefore limited by missing and incomplete data hence we were unable to compare the effect of access site (transseptal versus transapical) on left ventricular function and length of ICU stay. Since the outcomes were explored from published data and operator experience was not reported we were not able to assess the effect of operator experience on outcomes.

Future studies are needed to assess the mid- and long- term mortality for these two procedural approaches. One such study is the MITRAL (Mitral Implantation of TRAnscatheter vaLves)^16^ which is recruiting participants to establish the safety and feasibility of the Edwards SAPIEN XT and SAPIEN 3 device and delivery systems in patients with severe symptomatic calcific mitral valve disease with severe mitral annular calcification who are not candidates for standard mitral valve surgery. The study is also recruiting patients with failing surgical bioprosthesis or mitral rings who are not candidates for repeat mitral valve surgery. In the interim, further insight in the outcomes of these two procedural approaches can be explored from the United States Society of Thoracic Surgeons/American College of Cardiology TVT Registry, which will have the benefit of standardized entries that is less possible in reviews of case reports and series.

In summary, transcatheter mitral ViV and ViR using the two different procedural approaches appears to confer equal and reasonable 30-day outcomes. Patients in the transseptal group require more frequent closure of Iatrogenic ASD, whereas patients in the transapical group appear to have increased incidence of major bleeding.

## Ethical approval

Not applicable.

## Competing interests

None.

## Supplementary Materials

Supplementary file 1. Search starategy

Supplementary file 2 contains Table S1.
