# Supplementary material for: Outcomes following transcatheter transseptal versus transapical mitral valve-in-valve and valve-in-ring procedures
Source: J Cardiovasc Thorac Res. 2018 Dec 9;10(4):182–6. doi: 10.15171/jcvtr.2018.31 (PMC6335986; doi:10.15171/jcvtr.2018.31)
Supplement: Supplementary file 1 — Search starategy [file jcvtr-10-182-s001.pdf]

Search Strategy

PUBMED

((((((((transcatheter mitral valve in valve) OR transcatheter mitral valve in ring) OR transcatheter mitral valve implantation) OR transcatheter mitral) OR transcatheter mitral valve) OR transcatheter mitral valve replacement) OR transcatheter mitral valve repair) OR transcatheter mitral valve intervention) OR percutaneous transcatheter mitral valve

EMBASE

'transcatheter mitral valve in valve implantation'/exp OR 'transcatheter mitral valve in valve implantation' OR 'transcatheter mitral valve implantation' OR 'transcatheter mitral valve replacement'
