# Supplementary material for: Outcomes following transcatheter transseptal versus transapical mitral valve-in-valve and valve-in-ring procedures
Source: J Cardiovasc Thorac Res. 2018 Dec 9;10(4):182–6. doi: 10.15171/jcvtr.2018.31 (PMC6335986; doi:10.15171/jcvtr.2018.31)
Supplement: Supplementary file 2 — contains Table S1. [file jcvtr-10-182-s002.pdf]

Supplementary file 2

Table S1. Table showing co-morbidities, functional class, etiology of degeneration, type of valve, access site and valvular complications for patients with ViV and ViR

S.N	Author/Year	Previous other cardiac surgeries	Co-morbidities	NYHA class	Etiology of degeneration	Type of procedure	Type of valve	Access site	Valvular complications
1	Cheung et al[1]	CABG	CAD,COPD,CKD	-	Stenosis	MViV	26 mm Cribier 9000	Transapical	None
2	Webb et al[2]	-	-	IV	-	MViV	26 mm-Sapien XT	Transseptal	Valve embolization converted to open surgery
3	Webb et al[2]	-	-	IV	-	MViV	26 mm-Sapien XT	Transapical	None
4	Webb et al[2]	-	-	IV	-	MViV	26 mm-Sapien XT	Transapical	None
5	Webb et al[2]	-	-	IV	-	MViV	26 mm-Sapien XT	Transapical	None
6	Webb et al[2]	-	-	IV	-	MViV	23 mm-Sapien XT	Transapical	None
7	Webb et al[2]	-	-	IV	-	MViV	23 mm-Sapien XT	Transapical	None
8	Van Garsse et al[3]	-	IDDM,CKD	-	Stenosis	MViV	26 mm Sapien	Transapical	None
9	Seiffert et al[4]	-	A.FIB,CVA, GI Bleed, Breast Cancer	III	Stenosis	MViV	22 mm- Sapien	Transapical	None
10	Seiffert et al[4]	AVR, TAVR	CHF, CKD on HD,A.FIB,GI Bleed	III	Regurgitation	MViV	23 mm- Sapien	Transapical	None
11	Seiffert et al[4]	AVR	IDDM	III	Regurgitation	MViV	26 mm- Sapien	Transapical	None
12	Seiffert et al[4]	-	CAD, Breast Cancer	III	Regurgitation	MViV	26 mm- Sapien	Transapical	None
13	Nunez-Gil et al[5]	-	Severe P.HTN	IV	Mixed	MViV	23 mm- Sapien	Transapical	None
14	Montorfano et al[6]	-	-	-	Stenosis	MViV	26 mm-Sapien XT	Transapical	None
15	Poon et al[7]	CABG	CHF	IV	Stenosis	MViV	26 mm-Sapien	Transapical	None
16	Latib et al[8]	-	A.FIB	-	Stenosis	MViV	26 mm-Sapien XT	Transseptal	None
17	Elmariah et al[9]	CABG	CVA	-	Stenosis	MViV	26 mm- Sapien	Transapical	None
18	Theron et al[10]	-	-	-	Regurgitation	MViV	29 mm-Sapien XT	Transapical	None

19	Soon et al[11]	-	CKD	-	Regurgitation	MViV	29 mm-Sapien	Transapical	None
20	Bapat et al[12]	-	CHF	-	Stenosis	MViV	26 mm-Sapien	Transapical	None
21	Bapat et al[12]	-	IDDM, A.FIB	-	Regurgitation	MViV	26 mm-Sapien XT	Transapical	Valve embolization converted to open surgery
22	Schaefer et al[13]	AVR	COPD	III	Regurgitation	MViV	26 mm-Sapien	Transapical	None
23	Schaefer et al[13]	-	P.HTN	III	Regurgitation	MViV	26 mm-Sapien XT	Transseptal	None
24	Schaefer et al[13]	-	COPD, P.HTN	III	Regurgitation	MViV	26 mm-Sapien XT	Transseptal	None
25	Schaefer et al[13]	-	CKD on FD, P.HTN	III	Stenosis	MViV	26 mm-Sapien XT	Transseptal	None
26	Schaefer et al[13]	AVR	COPD	III	Regurgitation	MViV	23 mm-Sapien XT	Transseptal	None
27	Schaefer et al[13]	-	P.HTN	III	Stenosis	MViV	26 mm-Sapien XT	Transseptal	None
28	Michelena et al[14]	CABG, AVR	PAD	IV	Mixed	MViV	22 mm-Melody	Transseptal	None
29	Fassa et al[15]	-	-	-	Stenosis	MViV	26 mm-Sapien XT	Transseptal	Embolization of valve, Successful placement of second valve
30	Wilbring et al[16]	-	A.FIB, CVA, P.HTN	III	Mixed	MViV	29 mm-Sapien XT	Transapical	None
31	Wilbring et al[16]	-	A.FIB,CKD, P.HTN, IDDM	III	Mixed	MViV	29 mm-Sapien XT	Transapical	None
32	Wilbring et al[16]	-	CHF, CKD, CVA	III	Mixed	MViV	29 mm-Sapien XT	Transapical	None
33	Wilbring et al[16]	-	COPD,CVA, P.HTN	III	Mixed	MViV	26 mm-Sapien XT	Transapical	None
34	Wilbring et al[16]	-	A.FIB, COPD, P.HTN	III	Mixed	MViV	29 mm-Sapien XT	Transapical	None
35	Wilbring et al[16]	-	CHF, CVA, P.HTN	III	Mixed	MViV	26 mm-Sapien XT	Transapical	None
36	Wilbring et al[16]	-	CHF, CKD, CVA, P.HTN	III	Mixed	MViV	26 mm-Sapien XT	Transapical	None
37	Cheung et al[17]	CABG	-	-	Stenosis	MViV	26 mm- Cribier	Transapical	None
38	Cheung et al[17]	CABG	-	-	Stenosis	MViV	23 mm-Sapien	Transapical	None
39	Cheung et al[17]	AVR	-	-	Regurgitation	MViV	23 mm- Sapien	Transapical	None
40	Cheung et al[17]	-	-	-	Regurgitation	MViV	26 mm- Sapien	Transapical	None
41	Cheung et al[17]	-	-	-	Regurgitation	MViV	26 mm- Sapien	Transapical	None
42	Cheung et al[17]	CABG	-	-	Stenosis	MViV	23 mm- Sapien	Transapical	None

43	Cheung et al[17]	CABG	-	-	Mixed	MViV	23 mm- Sapien	Transapical	None
44	Cheung et al[17]	TVR	-	-	Regurgitation	MViV	26 mm- Sapien XT	Transapical	None
45	Cheung et al[17]	CABG	-	-	Stenosis	MViV	26 mm- Sapien XT	Transapical	None
46	Cheung et al[17]	CABG	-	-	Mixed	MViV	26 mm- Sapien XT	Transapical	None
47	Cheung et al[17]	CABG, TVR	-	-	Regurgitation	MViV	29 mm- Sapien XT	Transapical	None
48	Cheung et al[17]	CABG, AVR	-	-	Regurgitation	MViV	29 mm- Sapien XT	Transapical	None
49	Cheung et al[17]	CABG	-	-	Mixed	MViV	26 mm- Sapien	Transapical	None
50	Cheung et al[17]	-	-	-	Mixed	MViV	29 mm- Sapien XT	Transapical	None
51	Cheung et al[17]	-	-	-	Mixed	MViV	26 mm- Sapien	Transapical	None
52	Cheung et al[17]	TVR	-	-	Stenosis	MViV	23 mm- Sapien XT	Transapical	None
53	Cheung et al[17]	-	-	-	Stenosis	MViV	26 mm- Sapien	Transapical	None
54	Cheung et al[17]	CABG	-	-	Mixed	MViV	26 mm- Sapien	Transapical	None
55	Cheung et al[17]	AVR	-	-	Regurgitation	MViV	29 mm- Sapien XT	Transapical	None
56	Cheung et al[17]	TVR	-	-	Stenosis	MViV	26 mm- Sapien XT	Transapical	None
57	Cheung et al[17]	AVR	-	-	Mixed	MViV	29 mm- Sapien XT	Transapical	None
58	Rossi et al[18]	-	-	III	Regurgitation	MViV	26 mm-Sapien XT	Transapical	None
59	De Biasi et al[19]	CABG, TAVR	CAD,A.FIB, CKD	-	Regurgitation	MViV	26 mm-Sapien	Transapical	None
60	Bruschi et al[20]	-	-	-	-	MViV	26 mm-Sapien XT	Transseptal	Incomplete valve apposition requiring surgical valve replacement
61	Donofrio et al[21]	-	-	-	-	MViV	26 mm-Sapien XT	Transapical	None
62	Kliger et al[22]		CVA, CKD	IV	Mixed	MViV	24 mm- Melody	Transseptal	None
63	Kliger et al[22]		CVA, CKD, P.HTN	IV	Mixed	MViV	24 mm- Melody	Transseptal	None
64	Kliger et al[22]	CABG	P.HTN	III	Stenosis	MViV	24 mm- Melody	Transseptal	None
65	Kliger et al[22]	CABG, AVR		III	Regurgitation	MViV	25 mm- Melody	Transseptal	Valve embolization converted to open surgery
66	Kliger et al[22]	-	P.HTN	III	Mixed	MViV	25 mm- Melody	Transseptal	None
67	Kaneko et al[23]	AVR,	CAD, CKD, CVA	-	Stenosis	MViV	26 mm-Sapien	Transseptal	None

		CABG							
68	Duncan et al[24]	AVR	A.FIB, COPD, CAD	-	Stenosis	MViV	29 mm- Sapien XT	Transapical	None
69	Cerrillo et al[25]	AVR	-	-	Mixed	MViV	26 mm-Sapien	Transapical	None
70	Fuchs et al[26]	-	CAD	IV	Mixed	MViV	Sapien 3	Transseptal	None
71	Singh et al	-	-	-	Regurgitation	MViV	29 mm-Sapien XT	Transseptal	None
72	Chrissoheris et al[28]	-	-	IV	Stenosis	MViV	29 mm-Sapien XT	Transapical	None
73	Chaikriangkrai et al	-	CKD on HD		Stenosis	MViV	26 mm-Sapien XT	Transapical	None
74	Alli et al[30]	-	-	IV	Stenosis	MViV	29 mm-Sapien XT	Transapical	None
75	Baldizon et al[1]	CABG	A.FIB	-	-	MViV	29 mm-Sapien XT	Transapical	None
76	Schaefer et al[32]	-	P.HTN, PBC,CAD	III	Stenosis	MViV	25 mm-Lotus	Transapical	None
77	Schaefer et al[32]	-	P.HTN. Recurrent GI Bleeds	III	Regurgitation	MViV	27 mm-Lotus	Transapical	None
78	Schaefer et al[32]	-	P.HTN	III	Regurgitation	MViV	25 mm-Lotus	Transapical	None
79	Schaefer et al[32]		P.HTN, CKD, A.FIB	IV	Regurgitation	MViV	26 mm-Sapien 3	Transapical	None
80	Worku et al[34]	TVR	A.FIB	-	Regurgitation	MViV	26 mm-Sapien	Transapical	None
81	Worku et al[34]	CABG, AVR	CKD, A.FIB, CAD	-	Mixed	MViV	26 mm-Sapien	Transapical	None
82	Jochheim et al[35]	-	Hepatic Carcinoma, P.HTN, CHF	IV	Stenosis	MViV	23 mm-Sapien 3	Transseptal	None
83	Bruschi et al[6]	CABG	A.FIB, P.HTN,CKD, CHF	IV	Regurgitation	MViV	25 mm-DFM	Transapical	None
84	Ranney et al[37]	-	COPD, P.HTN	IV	Stenosis	MViV	26 mm-Sapien	Transapical	None
85	Ranney et al[37]	-	A.FIB, COPD	IV	Regurgitation	MViV	23 mm-Sapien XT	Transapical	None
86	Ranney et al[37]	AVR	A.FIB,CAD, CHF, P.HTN	IV	Stenosis	MViV	26 mm-Sapien	Transapical	None
87	Ranney et al[37]	CABG	A.FIB	IV	Regurgitation	MViV	29 mm-Sapien XT	Transapical	None
88	Schuler et al[38]	CABG, AVR	A.FIB	-	Mixed	MViV	22 mm-Melody	Transseptal	None

89	Akhras et al[39]	CABG	-	III	Regurgitation	MViV	29 mm-Sapien XT	Transapical	Valve thrombosis treated with anticoagulation
90	Cerillo et al[40]	AVR	CHF, CKD,CAD, CVA	IV	-	MViV	26 mm -Sapien	Transapical	None
91	Cerillo et al[40]	CABG	IDDM, CKD, CHF, PVD	III	-	MViV	26 mm- Sapien	Transapical	None
92	Cerillo et al[40]	TVR	COPD, CVA, A.FIB	IV	-	MViV	26 mm- Sapien	Transapical	None
93	Cerillo et al[40]	-	CHF, A.FIB	III	-	MViV	29 mm- Sapien	Transapical	None
94	Cerillo et al[40]	-	CKD	IV	-	MViV	26 mm- Sapien	Transapical	None
95	Cerillo et al[40]	-	NIDDM	III	-	MViV	26 mm- Sapien	Transapical	None
96	Cerillo et al[40]	TVR	MIDDM, A.FIB	III	-	MViV	26 mm- Sapien	Transapical	None
97	Cerillo et al[40]	AVR	CHF, A.FIB	IV	-	MViV	26 mm- Sapien	Transapical	None
98	Cerillo et al[40]	AVR	A.FIB	II	-	MViV	26 mm- Sapien	Transapical	None
99	Cerillo et al[40]	AVR, CABG	-	IV	-	MViV	26 mm- Sapien	Transapical	None
100	Cerillo et al[40]	-	CVA, A.FIB	IV	-	MViV	26 mm -Sapien	Transapical	None
101	Cerillo et al[40]	CABG	-	III	-	MViV	29 mm- Sapien	Transapical	None
102	Cerillo et al[40]	AVR,TVR	CKD	III	-	MViV	26 mm- Sapien	Transapical	None
103	Cerillo et al[40]	-	CVA, A.FIB, CKD	III	-	MViV	29 mm- Sapien	Transapical	None
104	Cerillo et al[40]	-	CHF	III	-	MViV	26 mm- Sapien	Transapical	None
105	Cerillo et al[40]	-	CHF	IV	-	MViV	23 mm- Sapien	Transapical	None
106	Cerillo et al[40]	-	COPD	III	-	MViV	29 mm- Sapien	Transapical	None
107	Nachum et al[41]	CABG	-	IV	Regurgitation	MViV	26 mm-Sapien	Transapical	None
108	Nachum et al[41]	CABG	-	IV	Regurgitation	MViV	29 mm-Sapien	Transapical	None
109	Nachum et al[41]	-	-	IV	Regurgitation	MViV	26 mm-Sapien	Transapical	None
110	Nachum et al[41]	-	-	IV	Regurgitation	MViV	29 mm-Sapien	Transapical	None
111	Nachum et al[41]	CABG	-	IV	Regurgitation	MViV	26 mm-Sapien	Transapical	None
112	Nachum et al[41]	CABG	-	IV	Regurgitation	MViV	26 mm-Sapien	Transapical	None
113	Nachum et al[41]	-	-	IV	Stenosis	MViV	26 mm-Sapien	Transapical	None
114	Nachum et al[41]	-	-	IV	Stenosis	MViV	26 mm-Sapien	Transapical	None
115	Nachum et al[41]	-	-	IV	Mixed	MViV	25 mm-Sapien	Transapical	None
116	Mick et al[42]	-	COPD,A.FIB, CVA	III	Regurgitation	MViV	23 mm-Sapien XT	Transapical	embolization and

									re-intervention MVIV using left atrial approach
117	Salun et al[43]	-	-	-	Regurgitation	MViV	29 mm-Sapien 3	Transapical	None
118	Rudzinski et al[44]	-	A,FIB, CKD, CVA	III	Stenosis	MViV	26 mm- Sapien 3	Transseptal	None
119	Eleid et al[45]	-	-	III	Stenosis	MViV	23 mm- Sapien	Transapical	Valve embolization converted to open surgery
120	Eleid et al[45]	-	-	III	Regurgitation	MViV	26 mm- Sapien	Transapical	Valve embolization converted to open surgery
121	Eleid et al[45]	-	-	III	Stenosis	MViV	23 mm- Sapien	Transapical	None
122	Eleid et al[45]	-	-	III	Regurgitation	MViV	26 mm- Sapien	Transseptal	None
123	Eleid et al[45]	-	-	III	Regurgitation	MViV	29 mm- Sapien XT	Transseptal	None
124	Eleid et al[45]	-	-	III	Regurgitation	MViV	29 mm- Sapien XT	Transseptal	None
125	Eleid et al[45]	-	-	III	Regurgitation	MViV	23 mm- Sapien XT	Transseptal	None
126	Eleid et al[45]	-	-	III	Regurgitation	MViV	29 mm- Sapien XT	Transseptal	None
127	Eleid et al[45]	-	-	III	Stenosis	MViV	26 mm- Sapien XT	Transseptal	None
128	Eleid et al[45]	-	-	III	Regurgitation	MViV	26 mm- Sapien XT	Transseptal	None
129	Eleid et al[45]	-	-	III	Stenosis	MViV	29 mm- Sapien XT	Transseptal	None
130	Eleid et al[45]	-	-	III	Regurgitation	MViV	29 mm- Sapien XT	Transseptal	Valve thrombosis treated with anticoagulation
131	Eleid et al[45]	-	-	III	Regurgitation	MViV	29 mm- Sapien XT	Transseptal	None
132	Eleid et al[45]	-	-	III	Regurgitation	MViV	29 mm- Sapien 3	Transseptal	None
133	Eleid et al[45]	-	-	III	Mixed	MViV	29 mm- Sapien 3	Transseptal	None
134	Eleid et al[45]	-	-	III	Stenosis	MViV	29 mm- Sapien 3	Transseptal	None
135	Eleid et al[45]	-	-	III	Stenosis	MViV	29 mm- Sapien 3	Transseptal	None
136	Eleid et al[45]	-	-	III	Regurgitation	MViV	29 mm- Sapien 3	Transseptal	None
137	Eleid et al[45]	-	-	III	Regurgitation	MViV	29 mm- Sapien 3	Transseptal	None
138	Eleid et al[45]	-	-	III	Stenosis	MViV	26 mm- Sapien	Transseptal	None
139	Eleid et al[45]	-	-	III	Regurgitation	MViV	29 mm- Sapien XT	Transseptal	None
140	Eleid et al[45]	-	-	III	Stenosis	MViV	29 mm- Sapien XT	Transseptal	None

141	Eleid et al[45]	-	-	III	Stenosis	MViV	29 mm- Sapien XT	Transseptal	None
142	Eleid et al[45]	-	-	III	Regurgitation	MViV	29 mm- Sapien XT	Transseptal	None
143	Eleid et al[45]	-	-	III	Stenosis	MViV	26 mm- Sapien XT	Transseptal	None
144	Eleid et al[45]	-	-	III	Mixed	MViV	26 mm- Sapien 3	Transseptal	None
145	Eleid et al[45]	-	-	III	Regurgitation	MViV	29 mm- Sapien 3	Transseptal	None
146	Eleid et al[45]	-	-	III	Regurgitation	MViV	29 mm- Sapien XT	Transseptal	None
147	Eleid et al[45]	-	-	III	Stenosis	MViV	26 mm- Sapien XT	Transseptal	None
148	Eleid et al[45]	-	-	III	Regurgitation	MViV	29 mm- Sapien XT	Transseptal	None
149	Eleid et al[45]	-	-	III	Regurgitation	MViV	23 mm- Sapien XT	Transseptal	None
150	Eleid et al[45]	-	-	III	Regurgitation	MViV	26 mm- Sapien XT	Transseptal	None
151	Eleid et al[45]	-	-	III	Regurgitation	MViV	29 mm- Sapien XT	Transseptal	None
152	Tada et al[46]	-	A.FIB	-	Regurgitation	MViV	23 mm-Sapien XT	Transapical	None
153	HE et al[47]	TVR	CKD, A.FIB	III	Mixed	MViV	29 mm-Sapien 3	Transapical	None
154	Herrmann et al[48]	CABG, TVR	A.FIB, COPD	III	Stenosis	MViV	29 mm-Sapien XT	Transseptal	None
155	Schaefer et al[13]	-	P.HTN	III	Regurgitation	MViR	29 mm- Sapien XT	Transapical	None
156	Schaefer et al[13]	-	P.HTN	III	Regurgitation	MViR	23 mm- Sapien XT	Transapical	None
157	Schaefer et al[13]	-	-	III	Regurgitation	MViR	29 mm- Sapien XT	Transapical	None
158	Schaefer et al[13]	-	P.HTN	III	Regurgitation	MViR	23 mm- Sapien XT	Transapical	None
159	Wunderlich et al[49]	-	CHF	III	Regurgitation	MViR	29 mm- Sapien XT	Transseptal	None
160	Maisano et al[50]	-	A.FIB	IV	Regurgitation	MViR	25 mm- Melody	Transapical	None
161	Attizzani et al[51]	TVR	-	III	Mixed	MViR	29 mm- Sapien XT	Transapical	None
162	Attizzani et al[51]	TVR		-	Regurgitation	MViR	26 mm- Sapien XT	Transapical	None
163	Latib et al[52]	-	-	IV	-	MViR	29 mm- DFM	Transapical	None
164	Latib et al[52]	-	-	IV	-	MViR	27 mm- DFM	Transapical	failure of implantation, retrieved and converted to surgery
165	Latib et al[52]	-	-	IV	-	MViR	25 mm- DFM	Transapical	None
166	Latib et al[52]	-	-	IV	-	MViR	25 mm- DFM	Transapical	None
167	Latib et al[52]	-	-	IV	-	MViR	29 mm- DFM	Transapical	None

168	Latib et al[52]	-		IV	-	MViR	27 mm- DFM	Transapical	None
169	Latib et al[52]	-	-	IV	-	MViR	29 mm- DFM	Transapical	failure of implantation
170	Latib et al[52]	-	-	IV	-	MViR	29 mm- DFM	Transapical	None
171	Lauterbach et al[53]	-	CKD, PAD, CAD	IV	Regurgitation	MViR	25 mm- Lotus	Transapical	None
172	Lauterbach et al[53]	CABG	CKD, A.FIB, CAD	IV	Regurgitation	MViR	27 mm-Lotus	Transapical	None
173	Wilbring et al[54]	TVR	IDDM, CKD, PVD	-	Regurgitation	MViR	26 mm- Sapien XT	Transapical	None
174	Eleid et al[45]	-	-	III	Mixed	MViR	26 mm- Sapein XT	Transseptal	None
175	Eleid et al[45]	-	-	III	Stenosis	MViR	23 mm- Sapien XT	Transseptal	None
176	Eleid et al[45]	-	-	III	Regurgitation	MViR	29 mm- Sapein XT	Transseptal	embolization converted to open surgery
177	Eleid et al[45]	-	-	III	Regurgitation	MViR	26 mm- Sapien XT	Transseptal	None
178	Eleid et al[45]	-	-	III	Stenosis	MViR	29 mm- Sapien 3	Transseptal	None
179	Eleid et al[45]	-	-	III	Regurgitation	MViR	29 mm- Sapien 3	Transseptal	None
180	Eleid et al[45]	-	-	III	Regurgitation	MViR	29 mm- Sapien XT	Transseptal	embolization converted to open surgery
181	Eleid et al[45]	-	-	III	Stenosis	MViR	26 mm- Sapien XT	Transseptal	None
182	Eleid et al[45]	-	-	III	Regurgitation	MViR	23 mm- Sapien XT	Transseptal	None
183	Pfeiffer et al[55]	CABG	CHF	-	Mixed	MViR	23 mm- Sapien XT	Transapical	None

Abbreviations: AVR: Aortic valve replacement, A.FIB: Atrial Fibrillation, CABG: Coronary artery bypass graft, CAD: Coronary artery disease, CHF: Congestive heart failure, CKD: Chronic Kidney disease, CVA: Cerebrovascular accident, COPD: Chronic obstructive pulmonary disease, DFM: Direct flow medical, GI: Gastrointestinal, HD: Hemodialysis, IDDM: Insulin dependent diabetes mellitus, MViV: Mitral valve-in-valve, MViR: Mitral valve-in-ring, PVD: Peripheral vascular disease, TAVR: Transcatheter aortic valve replacement, TVR: Tricuspid valve replacement

REFERENCES:

1. Cheung A, Webb JG, Wong DR, Ye J, Masson J-B, Carere RG, Lichtenstein SV. Transapical transcatheter mitral valve-in-valve implantation in a human. *Ann Thorac Surg* 2009;87:e18–20.

2. Webb JG, Wood DA, Ye J, Gurvitch R, Masson J-B, Rodés-Cabau J, Osten M, Horlick E, Wendler O, Dumont E, Carere RG, Wijesinghe N, Nietlispach F, Johnson M, Thompson CR, Moss R, Leipsic J, Munt B, Lichtenstein SV, Cheung A. Transcatheter valve-in-valve implantation for failed bioprosthetic heart valves. *Circulation* 2010;121:1848–1857.

3. Garsse LAFM van, Gelsomino S, Ommen V van, Lucà F, Maessen J. Emergency transthoracic transapical mitral valve-in-valve implantation. *J Interv Cardiol* 2011;24:474–476.

4. Seiffert M, Conradi L, Baldus S, Schirmer J, Knap M, Blankenberg S, Reichenspurner H, Treede H. Transcatheter mitral valve-in-valve implantation in patients with degenerated bioprostheses. *JACC Cardiovasc Interv* 2012;5:341–349.

5. Núñez-Gil IJ, Gonçalves A, Rodríguez E, Cobiella J, Marcos-Alberca P, Maroto L, Fernandez-Golfin C, Carnero M, Macaya C, Zamorano JL. Transapical mitral valve-in-valve implantation: a novel approach guided by three-dimensional transoesophageal echocardiography. *Eur J Echocardiogr* 2011;12:335–337.

6. Montorfano M, Latib A, Chieffo A, Moshiri S, Franco A, Grimaldi A, Alfieri O, Colombo A. Successful percutaneous anterograde transcatheter valve-in-valve implantation in the mitral position. *JACC Cardiovasc Interv* 2011;4:1246–1247.

7. Poon KKC, Clarke A, Luis SA, Wiemers P, Incani A, Scalia G, Tesar P, Raffel OC, Aroney CN, Walters DL. First Australian transapical mitral valve-in-valve implant for a failed mitral bioprosthesis: how to do it. *Heart Lung Circ* 2012;21:737–739.

8. Latib A, Ielasi A, Montorfano M, Maisano F, Chieffo A, Cioni M, Mussardo M, Bertoldi L, Shannon J, Sacco F, Covello RD, Figini F, Godino C, Grimaldi A, Spagnolo P, Alfieri O, Colombo A. Transcatheter valve-in-valve implantation with the Edwards SAPIEN in patients with bioprosthetic heart valve failure: the Milan experience. *EuroIntervention* 2012;7:1275–1284.

9. Elmariah S, Arzamendi D, Llanos A, Margey RJ, Inglessis I, Passeri JJ, Mehrotra P, Baker JN, Rosenfield K, Agnihotri AK, Vlahakes GJ, Palacios IF. First experience with transcatheter valve-in-valve implantation for a stenotic mitral prosthesis within the United States. *JACC Cardiovasc Interv* 2012;5:e13–14.

10. Théron A, Gariboldi V, Grisoli D, Maysou L, Jaussaud N, Morera P, Cuisset T, Quilici J, Thuny F, Riberi A, Avierinos J-F, Collart F. Three-dimensional transesophageal echocardiography assessment of a successful transcatheter mitral valve in valve implantation for degenerated bioprosthesis. *Echocardiography* 2013;30:E152–155.

11. Soon JL, Chua YL, Chao VT, Chiam PT, Ewe SH, Seetho VY, Lee CY, Tan SY, Koh TH, Sin KY. Asia's first successful minimally invasive transapical transcatheter mitral valve-in-valve implantation. *Ann Acad Med Singap* 2013;42:85–87.

12. Bapat VVN, Khaliel F, Ihleberg L. Delayed migration of Sapien valve following a transcatheter mitral valve-in-valve implantation. *Catheter Cardiovasc Interv* 2014;83:E150–154.

13. Schäfer U, Bader R, Frerker C, Schewel D, Thielsen T, Schmoeckel M, Kreidel F, Kuck K-H. Balloon-expandable valves for degenerated mitral xenografts or failing surgical rings. *EuroIntervention* 2014;10:260–268.

14. Michelena HI, Alli O, Cabalka AK, Rihal CS. Successful percutaneous transvenous antegrade mitral valve-in-valve implantation. *Catheter Cardiovasc Interv* 2013;81:E219–224.

15. Fassa A-A, Himbert D, Brochet E, Alkhoder S, Al-Attar N, Brun P-Y, Wolff M, Nataf P, Vahanian A. Emergency transseptal transcatheter mitral valve-in-valve implantation. *EuroIntervention* 2013;9:636–642.

16. Wilbring M, Alexiou K, Tugtekin SM, Sill B, Hammer P, Schmidt T, Simonis G, Matschke K, Kappert U. Transapical transcatheter valve-in-valve implantation for deteriorated mitral valve bioprostheses. *Ann Thorac Surg* 2013;95:111–117.

17. Cheung A, Webb JG, Barbanti M, Freeman M, Binder RK, Thompson C, Wood DA, Ye J. 5-year experience with transcatheter transapical mitral valve-in-valve implantation for bioprosthetic valve dysfunction. *J Am Coll Cardiol* 2013;61:1759–1766.

18. Rossi ML, Barbaro C, Pagnotta P, Cappai A, Ornaghi D, Belli G, Presbitero P. Transapical transcatheter valve-in-valve replacement for deteriorated mitral valve bioprosthesis without radio-opaque indicators: the "invisible" mitral valve bioprosthesis. *Heart Lung Circ* 2015;24:e19–22.

19. Biasi AR de, Wong S-C, Salemi A. Reoperative "valve-in-valve" transapical transcatheter mitral valve replacement in a high-risk patient with a recent transapical transcatheter aortic valve replacement and a degenerated bioprosthetic mitral valve. *J Thorac Cardiovasc Surg* 2014;148:e209–210.

20. Bruschi G, Botta L, Fratto P, Martinelli L. Failed valve-in-valve transcatheter mitral valve implantation. *Eur J Cardiothorac Surg* 2014;45:e127.

21. D'Onofrio A, Gallo M, Tarantini G, Cucchini U, Pittarello D, Gerosa G. An unexpected finding: stuck leaflet after transapical mitral valve-in-valve implantation. *JACC Cardiovasc Interv* 2014;7:e187–189.

22. Kliger C, Angulo R, Maranan L, Kumar R, Jelnin V, Kronzon I, Fontana GP, Plestis K, Patel N, Perk G, Ruiz CE. Percutaneous complete repair of failed mitral valve prosthesis: simultaneous closure of mitral paravalvular leaks and transcatheter mitral valve implantation - single-centre experience. *EuroIntervention* 2015;10:1336–1345.

23. Kaneko T, Swain JD, Loberman D, Welt FGP, Davidson MJ, Eisenhauer AC. Transjugular approach in valve-in-valve transcatheter mitral valve replacement: direct route to the valve. *Ann Thorac Surg* 2014;97:e161–163.

24. Duncan A, Davies S, Rosendahl U, Moat N. Consecutive transcatheter valve-in-valve implantations: the first in the aortic position, the second in the mitral position, in a patient with failing aortic and mitral bioprostheses. *BMJ Case Rep* 2014;2014.

25. Cerillo AG, Mariani M, Glauber M, Berti S. Simultaneous transapical paraprosthetic leak occlusion and valve-in-valve implantation into a degenerated mitral bioprosthesis. *J Heart Valve Dis* 2014;23:138–141.

26. Fuchs FC, Hammerstingl C, Sinning J-M, Mellert F, Werner N, Grube E, Nickenig G. Antegrade transcatheter mitral valve-in-valve implantation with combined atrial septal defect closure. *Clin Res Cardiol* 2016;105:460–462.

27. Singh GD, Smith TW, Boyd WD, Southard JA, Wong GB, Philip F, Low RI, Rogers JH. Complete Transcatheter Treatment of Degenerated Bioprosthetic Mitral Regurgitation: Transapical Paravalvular Leak Closure Followed by Transseptal Mitral Valve-in-Valve Replacement. *JACC Cardiovasc Interv* 2015;8:e229–231.

28. Chrissoheris M, Halapas A, Boumboulis N, Spargias K. Treatment of a Severely Degenerated Mitral Bioprosthesis with Transcatheter Valve-In-Valve Implantation. *Hellenic J Cardiol* 2015;56:347–350.

29. Chaikriangkrai K, Goswami R, Little S, Chang SM, Ramlawi B, Jhun HY, Kleiman N, Reardon MJ, Barker C. Bioprosthetic Mitral Transcatheter Transapical Valve-in-Valve Implantation for Mitral Stenosis in an End-Stage Renal Disease Patient. *J Card Surg* 2015;30:697–700.

30. Alli O, Booker O, Davies J. Emergent transcatheter mitral valve-in-valve implantation in a patient with cardiogenic shock secondary to a failed mitral bioprosthesis. *Catheter Cardiovasc Interv* 2016;87:1342–1346.

31. Baldizon I, Espinoza A, Kuntze T, Girdauskas E. Early transcatheter valve dysfunction after transapical mitral valve-in-valve implantation. *Interact Cardiovasc Thorac Surg* 2016;22:501–503.

32. Schaefer U, Conradi L, Lubos E, Deuschl F, Schofer N, Seiffert M, Treede H, Schirmer J, Reichenspurner H, Blankenberg S. First in human implantation of the mechanical expanding Lotus® valve in degenerated surgical valves in mitral position. *Catheter Cardiovasc Interv* 2015;86:1280–1286.

33. Schaefer A, Conradi L, Seiffert M, Lubos E, Blankenberg S, Reichenspurner H, Schaefer U, Treede H. Valve-in-Valve Procedures in Failing Biological Xenografts Using a Novel Balloon-Expandable Device: Experience in Aortic, Mitral, and Tricuspid Positions. *Thorac Cardiovasc Surg* 2016;64:366–373.

34. Worku B, Biasi AR de, Gulkarov I, Wong S-C, Salemi A. Transapical mitral valve-in-valve implantation for patients in cardiogenic shock. *Ann Thorac Surg* 2015;99:e103–105.

35. Jochheim D, Khandoga A, Bauer A, Baquet M, Theiss H, Schenzle J, Hausleiter J, Massberg S, Mehilli J. Transseptal Transcatheter Implantation of a Third-Generation Balloon-Expandable Valve in Degenerated Mitral Bioprosthesis. *JACC Cardiovasc Interv* 2015;8:e241–243.

36. Bruschi G, Cannata A, Barosi A, Colombo P, Soriano F, Nava S, Montrasio E, Botta L, Gagliardone MP, Klugmann S, Marco F De. Direct Flow valve-in-valve implantation in a degenerated mitral bioprosthesis. *EuroIntervention* 2016;11:1549–1553.

37. Ranney DN, Williams JB, Wang A, Gaca JG. Valve-in-Valve Transcatheter Valve Implantation in the Nonaortic Position. *J Card Surg* 2016;31:282–288.

38. Schuler A, Jones TK, Perpetua E, Aldea G, Reisman M, Mackensen GB. Two-in-One Using 3D: Mitral Paravalvular Leak Closure with Concomitant Transcatheter Valve-in-Valve Implantation. *J Cardiothorac Vasc Anesth* 2016.

39. Akhras N, Al Sergani H, Al Buraiki J, Fadel BM, Khaliel F, Al Allaf A, Al Amri M, Dahdouh Z. Thrombolytic Therapy as the Management of Mitral Transcatheter Valve-in-Valve Implantation Early Thrombosis. *Heart Lung Circ* 2016;25:e65–68.

40. Cerillo AG, Gasbarri T, Celi S, Murzi M, Trianni G, Ravani M, Solinas M, Berti S. Transapical Transcatheter Valve-in-Valve Implantation for Failed Mitral Bioprostheses: Gradient, Symptoms, and Functional Status in 18 High-Risk Patients Up to 5 Years. *Ann Thorac Surg* 2016;102:1289–1295.

41. Nachum ER, Raanani E, Segev A, Guetta V, Hai I, Shinfeld A, Fefer P, Ashraf H, Barabash I, Shalabi A, Spiegelstein D. Transapical Transcatheter Valve-in-Valve Implantation for Failed Mitral Valve Bioprosthesis. *Isr Med Assoc J* 2016;18:13–17.

42. Mick SL, Roselli EE, Kapadia S, Tuzcu EM, Krishnaswamy A, Svensson LG. Postoperative Migration of an Edwards-SAPIEN XT Mitral Valve-in-Valve Treated With Direct Vision Implantation During Beating-Heart Bypass. *Ann Thorac Surg* 2016;101:1182–1185.

43. Salaun E, Aldebert P, Jaussaud N, Spychaj J-C, Maysou LA, Collart F, Avierinos J-F, Casalta J-P, Cuisset T, Hubert S, Lambert M, Raoult D, Renard S, Habib G, Bonnet J-L. Early Endocarditis and Delayed Left Ventricular Pseudoaneurysm Complicating a Transapical Transcatheter Mitral Valve-in-Valve Implantation: Percutaneous Closure Under Local Anesthesia and Echocardiographic Guidance. *Circ Cardiovasc Interv* 2016;9.

44. Rudzinski PN, Dzielinska Z, Witkowski A, Konka M, Katarzyna K-L, Demkow M. Transcatheter Valve-in-Valve Implantation in a Degenerated Mitral Bioprosthesis Using a Trans-Septal Anterograde Approach and 3-D Transesophageal Echocardiography Guidance. *J Heart Valve Dis* 2016;25:90–92.

45. Eleid MF, Cabalka AK, Williams MR, Whisenant BK, Alli OO, Fam N, Pollak PM, Barrow F, Malouf JF, Nishimura RA, Joyce LD, Dearani JA, Rihal CS. Percutaneous Transvenous Transseptal Transcatheter Valve Implantation in Failed Bioprosthetic Mitral Valves, Ring Annuloplasty, and Severe Mitral Annular Calcification. *JACC Cardiovasc Interv* 2016;9:1161–1174.

46. Tada N, Enta Y, Sakurai M, Ootomo T, Hata M. Transcatheter valve-in-valve implantation for failed mitral prosthesis: the first experience in Japan. *Cardiovasc Interv Ther* 2017;32:82–86.

47. He C, Scalia G, Walters DL, Clarke A. Transapical Transcatheter Mitral Valve-in-Valve Implantation Using an Edwards SAPIEN 3 Valve. *Heart Lung Circ* 2017;26:e19–e21.

48. Herrmann HC, Szeto WY, Litt H, Vernick W. Novel use of perfusion balloon inflation to avoid outflow tract obstruction during transcatheter mitral valve-in-valve replacement. *Catheter Cardiovasc Interv* 2017.

49. Wunderlich NC, Kische S, Ince H, Bozdağ-Turan I. Transcatheter valve-in-ring implantation after a failed surgical mitral repair using a transseptal approach and a veno-arterial loop for valve placement. *Catheter Cardiovasc Interv* 2014;84:1202–1208.

50. Maisano F, Reser D, Pavicevic J, Nietlispach F, Gämperli O, Schmid M, Bettex D, Falk V. Successful first-in-man Melody transcatheter valve implant in a dehisced mitral annuloplasty ring transapical valve-in-ring implant. *EuroIntervention* 2014;10:961–967.

51. Attizzani GF, Cheung Tam C, Markowitz A. Transcatheter mitral valve-in-ring implantation in prohibitive surgical risk patients: Single center initial experience in the United States. *Catheter Cardiovasc Interv* 2016;88:E233–E238.

52. Latib A, Ruparelia N, Bijuklic K, Marco F De, Gatto F, Hansen L, Ozbek C, Greilach P, Bruschi G, Rieß F-C, Alfieri O, Colombo A, Schofer J. First-in-man transcatheter mitral valve-in-ring implantation with a repositionable and retrievable aortic valve prosthesis. *EuroIntervention* 2016;11:1148–1152.

53. Lauterbach M, Sontag B, Paraforos A, Friedrich I, Hauptmann K-E. Transcatheter valve-in-ring implantation of a repositionable valve system for treatment of severe mitral regurgitation. *Catheter Cardiovasc Interv* 2016;88:E183–E190.

54. Wilbring M, Kappert U, Matschke K. Transapical transcatheter valve-in-ring implantation for failed mitral valve repair in the absence of radiopaque markers. *J Thorac Cardiovasc Surg* 2015;149:e92–94.

55. Pfeiffer S, Gazdag L, Jessl J, Santarpino G. Transapical transcatheter valve-in-ring implantation following mitral annuloplasty. *J Card Surg* 2017;32:407–409.
